# Brain Abscesses in Domestic Ruminants: Clinicopathological and Bacteriological Approaches

**DOI:** 10.3390/microorganisms12071424

**Published:** 2024-07-13

**Authors:** Lucas Vinícius de Oliveira Ferreira, Thaís Gomes Rocha, Regina Kiomi Takahira, Renée Laufer-Amorim, Vânia Maria de Vasconcelos Machado, Márcio Garcia Ribeiro, Wanderson Adriano Biscola Pereira, José Paes Oliveira-Filho, Alexandre Secorun Borges, Rogério Martins Amorim

**Affiliations:** 1 Department of Veterinary Clinic, School of Veterinary Medicine and Animal Science, São Paulo State University (UNESP), Botucatu 18618-681, SP, Brazil; lv.ferreira@unesp.br (L.V.d.O.F.);; 2 Department of Veterinary Surgery and Animal Reproduction, School of Veterinary Medicine and Animal Science, São Paulo State University (UNESP), Botucatu 18618-681, SP, Brazil; 3 Department of Animal Production and Preventive Veterinary Medicine, School of Veterinary Medicine and Animal Science, São Paulo State University (UNESP), Botucatu 18618-681, SP, Brazil

**Keywords:** bacterial infection, cerebrospinal fluid, computer tomography, livestock, magnetic resonance imaging, neurological disease

## Abstract

Brain abscesses in ruminants often arise from primary infection foci, leading to an unfavorable prognosis for affected animals. This highlights the need for comprehensive studies on brain abscesses across different ruminant species. We retrospectively investigated medical records of epidemiological, clinical, neuroimaging, anatomopathological, and bacteriological findings in six ruminants (three goats, two cows, and one sheep) diagnosed with brain abscesses. All animals studied were female. Apathy (50%), compulsive walking (33%), decreased facial sensitivity (33%), head pressing (33%), seizures (33%), semicomatous mental status (33%), strabismus (33%), unilateral blindness (33%), and circling (33%) represented the most common neurologic signs. Leukocytosis and neutrophilia were the main findings in the hematological evaluation. Cerebrospinal fluid (CSF) analysis revealed predominant hyperproteinorrachia and pleocytosis. In three cases, computed tomography or magnetic resonance imaging were used, enabling the identification of typical abscess lesions, which were subsequently confirmed during postmortem examination. Microbiological culture of the abscess samples and/or CSF revealed bacterial coinfections in most cases. Advanced imaging examinations, combined with CSF analysis, can aid in diagnosis, although confirmation typically relies on postmortem evaluation and isolation of the causative agent. This study contributes to clinicopathological aspects, neuroimages, and bacteriological diagnosis of brain abscesses in domestic ruminants.

## 1. Introduction

Brain abscesses in ruminants typically originate from primary infectious foci, and they can spread through hematogenous routes [1], lymphatic pathways [2,3], though peripheral nerves, direct penetrating injuries [4,5], extension from adjacent suppurative lesions (e.g., sinusitis, dehorning, otitis) [5,6], and nasopharyngeal infections [3].

The main agent isolated from these abscesses is *Trueperella pyogenes* [1]. However, other pathogens have also been described in association with infections in the central nervous system (CNS) of ruminants, such as *Fusobacterium necrophorum*, *Staphylococcus* sp., *Escherichia coli*, *Streptococcus* sp., *Actinomyces* sp., *Listeria monocytogenes*, and *Corynebacterium pseudotuberculosis* [5,6,7,8,9,10].

The clinical manifestations vary and depend on the number, size, location, and rate of development of the brain abscess [5]. Typically, clinical presentations have a slow onset and worsen progressively as the abscess increases and affects other regions [6].

The routine diagnosis is based on clinical signs, neurological examination, and cerebrospinal fluid (CSF) analysis. Advanced imaging exams, such as computed tomography (CT) or magnetic resonance imaging (MRI), have proven useful for diagnosing clinical conditions in cattle [1,11,12,13,14] and goats [7,15]. However, confirmation is often achieved through postmortem examination [6] and microbiological identification [8].

The prognosis is unfavorable due to unsatisfactory outcomes from treatment with anti-inflammatories and broad-spectrum antibiotic therapy, mainly because of the difficulty in achieving therapeutic concentrations on the lesional foci [1,6]. Despite infections of the CNS being considered common in ruminants and of great significance for animal health and production [9,16,17], there is a lack of comprehensive studies regarding brain abscesses in different species of ruminants. Considering this scenario, we retrospectively examined the epidemiological, clinical, neuroimaging, postmortem, and microbiological aspects of domestic ruminants diagnosed with brain abscesses.

## 2. Materials and Methods

### 2.1. Local Study and Contextualization

All clinical records from 2011 to 2022 of ruminants suspected of having brain abscesses were retrospectively reviewed. The ruminants were referred to the Large Animal Clinic Service of the Teaching Veterinary Hospital in Botucatu County (22°53′09′′ S, 48°26′42′′ W), State of São Paulo, Brazil. The inclusion criteria for this study were postmortem diagnosis of brain abscesses with microbiological isolation.

### 2.2. Epidemiological, Clinical, and Postmortem Data

Epidemiological aspects encompassed species (cattle, goats, and sheep), breed, gender, and age. Clinical aspects (duration of the disease, results of physical and laboratory examinations, length of hospitalization, and progression) were extracted from veterinary reports documented in the medical records. Blood tests, CSF analysis, necropsy, and microscopic examinations were routinely performed.

### 2.3. CSF Samples

CSF samples were obtained from the animals under sedation or general anesthesia using a sterile spinal needle inserted into either the atlantooccipital or lumbosacral sites. The CSF samples were placed in three sterile tubes and promptly sent for laboratory analysis. Protein levels in the CSF were determined using a specific biochemical kit (Bioprot U/LCR—Bioclin, Belo Horizonte, Brazil). Cell counting was performed in a Neubauer chamber, followed by cytocentrifugation for differential analysis. Complete blood counts were conducted using pocH-100iV Diff—Sysmex, Koibe, Japan). All obtained data were analyzed descriptively.

### 2.4. MRI and CT

MRI imaging was conducted using a 0.25 Tesla scanner (Vet-MR Grande, Esaote, Genoa, Italy), including T1-weighted, T2-weighted, and fluid attenuation inversion recovery (FLAIR) sequences in the sagittal, transverse, and dorsal planes. CT scans were performed using helical equipment SCT-7800TC (Shimadzu; Kyoto, Japan). The images were evaluated through a medical digital imaging system (PACS, Synapse, Fuji Medical System; Tokyo, Japan).

### 2.5. Microbiological Culture and Identification of Microorganisms

Fragments of the brain abscess collected during the postmortem examination and CSF samples were inoculated onto defibrinated bovine blood agar (5%) and MacConkey agar media for microbiological culture and incubated at 37 °C for 72 h. Simultaneously, the same samples were subjected to microaerophiles (5% CO_2_) and anaerobic cultures on bovine blood agar and incubated at 37 °C for 120 h. Identification of the microorganisms was performed based on morphotintorial, biochemical, and phenotypic aspects [18].

In Vitro Susceptibility Test

Isolates were subjected to in vitro susceptibility testing (disk diffusion method), according to the Clinical Laboratory Standards Institute—CLSI guidelines [19], using 15 antimicrobials belonging to nine groups, as follows: (1) aminoglycosides (amikacin 30 μg, gentamicin 10 μg), (2) beta-lactams and derivatives (amoxicillin 10 μg; ampicillin 10 μg; ceftiofur 30 μg; penicillin 10 μg), (3) fluoroquinolones (enrofloxacin 5 μg), (4) lincosamides (clindamycin 2 μg), (5) macrolides (azithromycin 15 μg, erythromycin 15 μg); (6) phenicols (chloramphenicol 30 μg, florfenicol 10 μg), (7) rifamycins (rifampicin 5 μg), (8) sulfonamides (sulfamethoxazole 25 μg), and (9) tetracyclines (tetracycline, 30 μg).

## 3. Results

### 3.1. Animals and Epidemiological Data

Six ruminants meet the inclusion criteria. The epidemiological findings of six domestic ruminants studied included one sheep (case 1), two cows (cases 2 and 3), and three goats (cases 4, 5, and 6) (Table 1). All were females, with ages ranging from one month to 48 months (mean = 21 months). The duration of the disease on the breeding farms of animals until clinical evaluation ranged from two to 60 days (mean = 18 days). The hospitalization period varied from one day to 21 days (mean = six days). Lethality was observed in all the animals.

### 3.2. Clinical Signs

During the initial physical examination, the sheep (Case 1) exhibited a heart rate, respiratory rate, and rectal temperature of 136 beats per minute (bpm), 36 movements per minute (mpm), and 38.5 °C, respectively.

For the cattle (Case 2 and 3), the mean values for heart rate, respiratory rate, and rectal temperature were 54 bpm, 26 mpm, and 38.9 °C, respectively. Notably, Case 3 displayed marked bradycardia with a heart rate of 36 bpm. In the case of the goats (Cases 4, 5, and 6), the mean values were 110 bpm, 36 mpm, and 38 °C, respectively.

**Figure 1:**
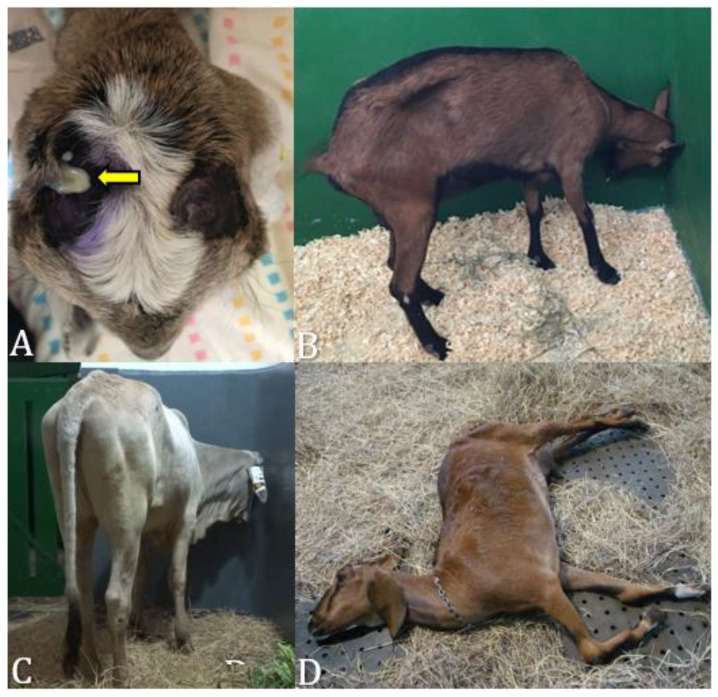
Clinical manifestation of animals affected by brain abscesses. (**A**) Case 4: a goat presenting purulent discharge from the horn (yellow arrow) after dehorning. (**B**,**C**) Case 5 and 3: a goat and a cow presenting head pressing, respectively. (**D**) Case 6: a goat presenting opisthotonus.

In the animals of the present study, 50% (3/6) exhibited dehydration ranging from 5% to 10%, 50% (3/6) showed congested mucous membranes and 50% (3/6) pink mucous membranes. Additionally, 50% (3/6) displayed ruminal hypomotility, 33% (2/6) had ruminal atony, and 17% (1/6) presented with purulent discharge from the horn (Figure 1A). Upon arrival for treatment, 50% (3/6) of the animals were in lateral recumbency, and 50% (3/6) were standing.

In the neurological evaluation, the affected animals exhibited various alterations (Table 1). Regarding mental status changes, 50% (3/6) were apathetic, 17% (1/6) were alert, and 33% (2/6) were in a semicomatose mental state. In terms of behavioral changes, 33% (2/6) exhibited compulsive walking, 17% (1/6) presented paresis, 33% (2/6) circling, 33% (2/6) (Videos S1 and S2) head pressing (Figure 1B,C), 33% (2/6) seizures, 17% (1/6) lateral head tilting, 17% (1/6) excessive vocalization, 17% (1/6) opisthotonus (Figure 1D), 33% (2/6) unilateral blindness, 17% (1/6) amaurosis, 17% (1/6) fasciculations, 17% (1/6) ptosis (ear, eyelid, and lip), 33% (2/6) decreased facial sensitivity, 17% (1/6) difficulty in grasping, chewing, and swallowing food, 17% (1/6) nystagmus, and 33% (2/6) strabismus (lateral, dorsomedial, medial, ventrolateral).

### 3.3. Laboratory Findings

Leukocytosis with neutrophilia was the predominant hematological abnormality observed in this study (Table 2). The CSF analysis obtained from the animals is described in detail in Table 3. In cases 1 and 4, the collection of CSF in the atlantooccipital region was unsuccessful. Thus, the decision was made to proceed with the collection in the lumbosacral region.

### 3.4. Image Findings

**Figure 2:**
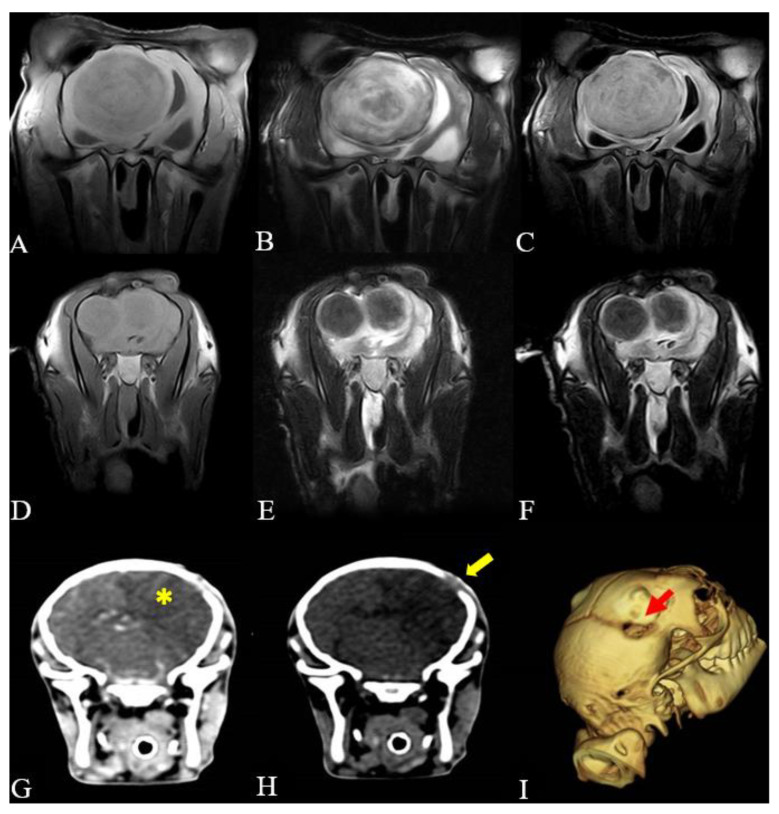
Magnetic resonance imaging and computed tomography of the brain. (**A**–**C**) Case 3: Postmortem MRI images at the level of the temporal/parietal lobes. (**A**) Transverse T1W image. A well-circumscribed region of hypointensity is present with a heterogeneous appearance (**B**) Transverse T2W image. The region of hypointensity seen on the T1W image in (**A)** is surrounded by a contiguous hyperintensity rim. (**C**) Transverse fluid attenuation inversion recovery (FLAIR) image. Notice that the lateral ventricles are compressed, and the circumscribed hypointensity region is poorly defined. This is consistent with the MRI features of a mature abscess. (**D**–**F**) Case 5: Antemortem MRI images at the level of the temporal/parietal lobes. (**D**) Transverse T1W image. Notice dual adjacent well-circumscribed hypointense structures throughout the white matter with a corresponding midline shift and protrusion into the right hemisphere of the brain. (**E**) Transverse T2W image. Ring enhancement surrounding a hypointense structure is highly defined. (**F**) Transverse fluid attenuation inversion recovery (FLAIR) image. The enhancement of the ring surrounding the hypointense structures is less defined, the meninges are hyperintense, and there is edema adjacent to the structures. MRI T1W, T2W, and FLAIR images correspond to a mature brain abscess. (**G**–**I**) Case 4: (**G**) Antemortem tomographic image demonstrating a hypodense region (10.00 HU) in the right cerebral cortex (yellow asterisk). (**H**) Tomographic image revealing a bone discontinuity in the cranial vault (yellow arrow). (**I**) Three-dimensional reconstruction of the skull, highlighting the bone discontinuity in the cranial vault (red arrow).

In cases 3 and 5, MRI imaging was performed, and in case 4, a CT scan was conducted. In case 3, the examination was conducted postmortem, and the obtained images revealed the presence of a well-defined circular structure with a heterogeneous appearance, predominantly hypointense relative to the cerebral parenchyma in T1 and FLAIR, and hyperintense in T2, measuring 7.7 × 6.8 × 6.7 cm, occupying almost the entire right hemisphere and compressing the hypothalamus/thalamus, left cerebral parenchyma, brainstem and cerebellum (Figure 2A–C). Additionally, loss of cerebral sulci and gyri, cerebellar changes, and significant dilation of the lateral ventricles and mesencephalic aqueduct were observed. In case 5, MRI imaging was performed antemortem under general anesthesia and revealed two well-circumscribed hypointense structures adjacent to the white matter with a corresponding midline shift and protrusion into the right hemisphere of the brain (Figure 2D–F). In case 4, the CT scan was performed antemortem under general anesthesia, and it was possible to identify an extensive hypodense area in the right frontal, parietal, and temporal lobes, causing a midline shift towards the left cerebral hemisphere. There was slight enhancement after contrast administration, along with bone discontinuity of the ipsilateral temporal bone (Figure 2G–I). Due to the owner’s compliance and unfavorable prognosis, both animals were euthanized.

### 3.5. Postmortem Evaluation and Microbiological Isolation

**Figure 3:**
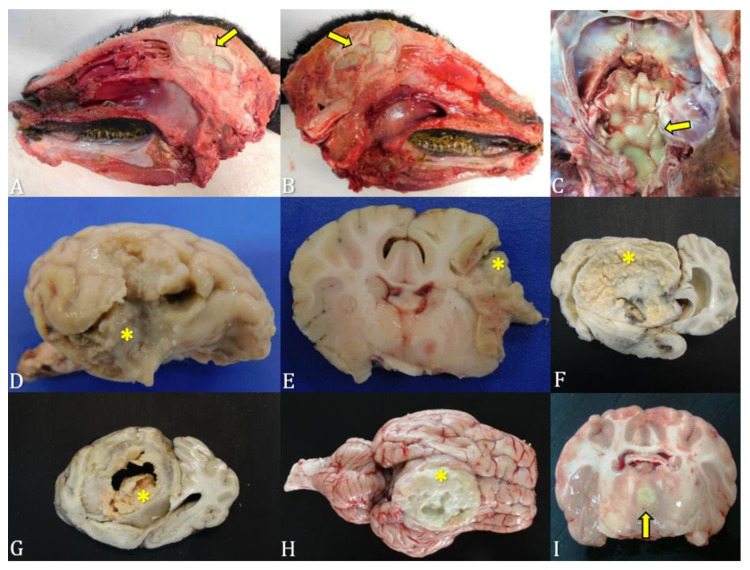
Identification of brain abscesses in postmortem examination in domestic ruminants. (**A**,**B**) Case 1: abscess located in the prosencephalon (yellow arrows). (**C**) Case 2: Abscess in the *rete mirabile* (yellow arrows). (**D**,**E**) Case 3: Abscess in the prosencephalon (yellow asterisks). (**F**,**G**) Case 4—abscess occupying the entire right cerebral hemisphere (yellow asterisks). (**H**) Case 5—Abscess in the prosencephalon (yellow asterisk). (**I**) Case 6—Abscess located in the mesencephalic region (yellow arrow).

**Figure 4:**
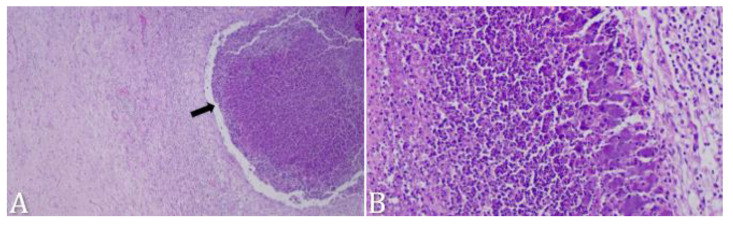
Photomicrographs of histological sections from case 1 with a brain abscess in the prosencephalon of a sheep. Hematoxylin and eosin (H&E) stained. (**A**) Delimited is of liquefaction, characterized by a capsule and a space filled with degenerated neutrophils (black arrow). (**B**) Higher magnification of (**A**). Notice the degenerated neutrophils that compose the abscess.

In the postmortem examination, the abscess was identified in the prosencephalon in 50% (3/6) of the animals (Figure 3A,B,D,E,H), in the *rete mirabile* in 17% (1/6) (Figure 3C), involving the entire right hemisphere in 17% (1/6) (Figure 3F,G), and in the mesencephalon in 17% (1/6) (Figure 3I). In histopathological analyses, inflammatory processes composed of mononuclear and polymorphonuclear cells, associated with areas of necrosis (Figure 4A,B) were predominantly observed.

Samples of caseopurulent materials from the abscesses were submitted to microbiological culture, and the results are shown in Table 4. Furthermore, in cases 2 and 5, it was also possible to isolate the agents from CSF (Table 4). In vitro, antimicrobial susceptibility profile was performed using isolates from cases 4 and 6 (Table 5).

## 4. Discussion

Neurological diseases hold significant clinical importance in production animals, leading to considerable economic losses and impacts on animal welfare [17,23]. Bacterial infections play a crucial role in this context, potentially leading to the formation of brain abscesses. However, determining the primary cause for the formation of the abscess is not always feasible [24].

In the present study, in case 4, the primary cause could be identified based on the clinical history and the observation of purulent discharge from the right horn after the hot iron dehorning. The nonjudicious execution of this procedure, with high temperatures, may render the bone tissue more susceptible to pathogen infection, thereby promoting abscess formation, as evidenced in the postmortem examination.

Brain abscesses has been described in animals younger than 12 months old [12,15,25,26] and in older animals aged five to six years [26,27]. The age range of the animals in the present study ranged from one to 48 months, suggesting that the disease can occur in both young and mature ruminants.

All observed cases in this study were females, as previously reported [12,15,26,27], probably due to the fact that females represent the majority of the herds used for food production. However, there are also descriptions of the disease in males [1,28]. It is believed that the aggressive behavior of males may contribute to cranial traumas and head injuries, potentially triggering infectious processes [29,30]. Additionally, the use of nose rings for more effective restraint of male animals may increase the predisposition to the occurrence of this condition [31].

The clinical manifestations observed in this study were similar to those previously reported [4,6,27,28]. The clinical signs are widely variable, a fact that can be attributed to the infectious/inflammatory process, neurological disorder based on the regions affected by the abscess, or increased intracranial pressure, which are common to various diseases affecting the CNS. In case 3, the bovine exhibited bradycardia (36 bpm), similar to the findings reported by Braun et al. [1], who identified a bull with a pituitary abscess presenting a heart rate of 32 bpm during the physical examination. According to the authors, it is suggested that changes in the nuclei of the vagus nerve in the *medulla oblongata* may be responsible for triggering the observed vagotonic bradycardia [1].

Despite some studies reporting normal leukocyte values [1,12], the main hematological findings of this study were leukocytosis and neutrophilia, results consistent with previous descriptions [7,14]. These findings are indicative of an infectious/inflammatory process that may be associated with the presence of brain abscess.

The most notable changes in the CSF were the consistent presence of hyperproteinorrachia in all cases analyzed, as previously reported [1]. Moreover, the identification of mixed pleocytosis in three cases and neutrophilic pleocytosis in one case aligns with previous observations [1,14,27], confirming the presence of an inflammatory process in the CNS. In cases where the abscess develops internally within the cerebral parenchyma, abnormalities in the CSF may not be observed [7]. The abnormalities can be identified depending on the extent of the inflammatory process in the meninges or the rupture of the abscess [32].

The imaging findings in the present animals studied are in accordance with previous reports [7,11,12,15]. In humans, imaging is considered the basis for diagnosis, enabling early identification of the abscess, including its size, number, and location [33,34]. Since the implementation of advanced imaging, a reduction in mortality has been observed, resulting in significant improvements in prognosis [35,36]. In the present study, we observed a relation between the images and clinical changes, suggesting the presence of brain abscesses in the animals. This condition was subsequently confirmed during the postmortem examination. MRI and CT proved highly valuable when conducted antemortem, contributing to the diagnosis and prognosis of the animals. Therefore, these imaging methods should be considered, when available, in cases of suspected CNS diseases in ruminants.

Nevertheless, in most cases, a definitive diagnosis of brain abscess is obtained through postmortem examination [6]. Abscesses can develop in any region of the CNS, with the affected site being associated with the primary source of infection. However, the most affected site in this study was the prosencephalon, in accordance with other studies [16,37]. Our histopathological findings resemble previous descriptions [15,16,27] and support the macroscopic observations of abscess formation.

The microbiological examination of the samples from the abscess secretions identified coinfections between bacteria in most cases, as observed in other species [38,39]. *Escherichia coli* was the most frequent (50%) agent isolated among the majority of animals studied.

*Trueperella pyogenes* is an actinomycete considered the main agent isolated from brain abscesses [1,6]. Nonetheless, the pathogen was identified in 33% (2/6) of the cases reported herein, affecting one sheep and one cow. In addition, the agent has also been reported in abscesses in goats [8,15], reinforcing the opportunistic behavior and propensity of the pathogen as a cause of brain abscesses in domestic ruminants [40].

*Corynebacterium pseudotuberculosis*, another actinomycete, was identified in 33% (2/6) of the animals, as previously reported [8]. The actinomycetes are characterized by opportunistic behavior, causing pyogranulomatous reactions in animals [40,41], including in CNS, and reinforces that these groups of bacteria should be considered as primary agents of brain abscesses in domestic ruminants. Other bacteria have also been isolated from brain abscesses of domestic ruminants, including *Corynebacterium ulcerans* [26], *Fusobacterium necrophorum* [12], *Streptococcus* sp., *Actinomyces* sp. [8], and *Acinetobacter baumanni* [42], highlighting the importance of microbiological identification in diagnostic approaches of brain abscesses in domestic ruminants.

Microbiological isolation from CSF was feasible in 33% (2/6) of cases, agreeing with the findings of Camara et al. [43], who identified *Trueperella pyogenes* in a cattle with a pituitary abscess in one of the CSF samples. Microbiological analysis of CSF is a valuable tool for CNS diseases, as bacteria can cross the blood-brain barrier and colonize the CSF [1], enabling antemortem isolation of the agent and determining the antimicrobial susceptibility profile for the implementation of a potential treatment. Although the treatment is generally considered inefficient, there is a report of favorable results in a bovine with pituitary abscess using a combination of amoxicillin, gentamicin, and flunixin meglumine [1].

A convenient number of animals studied, a lack of conducting advanced imaging examinations (MRI and CT) on all animals sampled, and no in vitro antimicrobial susceptibility profile testing in all bacteria isolated may be considered limitations of the current study.

## 5. Conclusions

Overall, six domestic ruminants with brain abscesses retrospectively studied reveal a predominance of cases in females, showing leukocytosis and neutrophilia, identification mainly of *E. coli* and actinomycetes (*T. pyogenes* and *C. pseudotuberculosis*), and a wide variety of neurological signs, likely due to different foci of bacterial lesions in brain tissue. The combination of clinical and epidemiological data, hematological, microbiological, and advanced imaging approaches may contribute to early diagnosis and establishment of prognosis in brain abscesses of domestic ruminants.

## Figures and Tables

**Table 1:** Epidemiological findings and neurological examination of the six domestic ruminants diagnosed with brain abscess.

Case	Species	Breed	Age	Gender	Weight	Period of Evolution of Signs until Care	Period of Hospitalization Until Death/Euthanasia	Main Findings in Neurological Examination
1	Sheep	Suffolk	24 months	F	50 kg	13 days	1 day	Lateral recumbency, semicomatous state, unilateral blindness, and seizures
2	Cow	Dutch	6 months	F	180 kg	2 days	7 days	Difficulty in grasping, chewing, and swallowing food, and lateral strabismus
3	Cow	Nelore	15 months	F	253 kg	60 days	3 days	Apathy, compulsive and circling gait, paresis, head pressing, unilateral blindness, auricular ptosis, decreased facial sensitivity, and ventrolateral strabismus
4	Goat	Anglo Nubian	1 month	F	5.8 kg	4 days	4 days	Lateral recumbency, semicomatous state, lateral head tilt, decreased facial sensitivity, and seizures
5	Goat	Alpine	36 months	F	45 kg	30 days	21 days	Apathy, compulsive and circling gait, head pressing, excessive vocalization, and amaurosis
6	Goat	Anglo Nubian	48 months	F	45 kg	3 days	2 days	Apathy, lateral recumbency, opisthotonus, fasciculations, and horizontal and vertical nystagmus

F: Female.

**Table 2:** Hematological values in six domestic ruminants diagnosed with brain abscess.

Parameters	Case 1	Sheep Reference	Case 2	Case 3	Bovine Reference	Case 4	Case 5	Case 6	Goat Reference
Haematocrit (%)	24	27–45	23	25	24–46	26	25	44	22–38
Red blood cells (10^6^/μL)	10.28	9–15	5.02	6.24	5–10	NP	NP	16.95	8–18
Hemoglobin (g/dL)	8.6	9–15	7.1	8.0	8–15	9.1	10.5	15.6	8–12
Total protein (g/dL)	8.6	6–7.5	8.6	7.2	7.0–8.5	6.6	9.6	10	6–7.5
Fibrinogen (mg/dL)	1000	100–500	400	200	300–700	1200	200	1000	100–400
Total leukocytes (/μL)	31,800	4000–12,000	15,400	12,200	4000–12,000	23,000	12,600	26,600	4000–13,000
Neutrophils (/μL)	29,900	700–6000	6300	5500	600–4000	17,500	8700	23,400	1200–7200
Lymphocytes (/μL)	1900	2000–9000	8300	6000	2500–7500	4100	3500	2900	2000–9000
Monocytes (/μL)	0	0–750	800	200	25–840	1400	100	300	0–550
Eosinophils (/μL)	0	0–1000	0	400	0–2400	0	300	0	50–650
Basophils (/μL)	0	0–300	0	100	0–200	0	0	0	0–120

NP: Not performed; Reference values: Kramer [20].

**Table 3:** Analysis of cerebrospinal fluid in six domestic ruminants diagnosed with brain abscess.

Identification	Cerebrospinal Fluid (CSF)			
	Color	Aspect	Protein (mg/dL) Ovine (8–70) Bovine (20–40) Caprine (24–40)	Cellularity (<10 cells/µL)
Case 1	NP	NP	NP	NP
Case 2	Colorless	Clear	43.6	100 cells/µL: Mixed pleocytosis—Predominance of mononuclear cells (50%), followed by neutrophils (27%), typical lymphocytes (19%), macrophages (4%), and rare eosinophils. Presence of cytophagocytosis and free and phagocytosed round structures
Case 3	Colorless	Clear	108.1	95 cells/µL: Mixed pleocytosis—Predominance of mononuclear cells (46%), followed by typical lymphocytes (37%), macrophages (12%), and neutrophils (5%). Presence of clusters of mononuclear cells
Case 4	Xanthochromic	Clear	783.7	149 cells/µL: Mixed pleocytosis—Predominance of neutrophils (57%) followed by mononuclear cells (35%), typical lymphocytes (6%) and macrophages (2%)
Case 5	Colorless	Clear	44.4	1 cell/µL: Predominance of typical lymphocytes (82%), followed by mononuclear cells (14%), neutrophils (2%), and macrophages (2%)
Case 6	Whitish	Turbidity	100	10.450 cells/µL: Neutrophilic pleocytosis—Predominance of neutrophils (79%), followed by mononuclear cells (12%) and typical lymphocytes (9%). Presence of leukophagocytosis

Protein reference: Smith et al. [21]; Cellularity reference: Dore and Smith [6], Scott [22]; Pleocytosis reference: Schöb et al. [17]; NP: Not performed.

**Table 4:** The location of the abscesses and microbiological identification of six domestic ruminants diagnosed with brain abscess.

Case	Site	Isolation	
		Brain Abscesses	CSF
1	Prosencephalon	*Trueperella pyogenes*, *Pasteurella multocida*, and *Escherichia coli*	NP
2	*Rete mirabile*	*Escherichia coli* and *Streptococcus* spp.	*Escherichia coli* and *Streptococcus* spp.
3	Right cerebral hemisphere	*Trueperella pyogenes* and *Streptococcus* spp.	Negative
4	Prosencephalon	*Escherichia coli*, *Enterobacter* sp., and *Staphylococcus* sp.	Negative
5	Prosencephalon	*Corynebacterium pseudotuberculosis*	*Corynebacterium pseudotuberculosis*
6	Mesencephalon	*Corynebacterium pseudotuberculosis*	Negative

CSF: Cerebrospinal fluid; NP: Not performed.

**Table 5:** In vitro antimicrobial susceptibility profile of two goats diagnosed with brain abscess.

Case	Pathogen	AMI	AMO	AMP	AZI	CEF	CLI	ENR	ERY	FLO	GEN	PEN	RIF	SUL	TET
	*Escherichia coli*	S	S	R	NP	S	R	NP	NP	NP	S	NP	R	NP	R
4	*Enterobacter* sp.	S	S	R	NP	S	R	NP	NP	NP	S	NP	R	NP	R
	*Staphylococcus* sp.	S	R	R	NP	I	R	NP	NP	NP	S	NP	R	NP	I
6	*Corynebacterium pseudotuberculosis*	NP	NP	S	S	S	NP	S	S	S	NP	S	S	S	NP

AMI: Amikacin; AMO: Amoxicillin; AMP: Ampicillin; AZI: Azithromycin; CEF: Ceftiofur; CLI: Clindamycin; ENR: Enrofloxacin; ERY: Erythromycin; FLO: Florfenicol; GEN: Gentamicin; PEN: Penicillin; RIF: Rifampicin; SUL: Sulfamethoxazole; TET: Tetracycline; S: Sensitive; R: Resistant; I: Intermediate, NP: Not performed.

## Data Availability

Data are contained within the article.

## Supplementary Materials

The following supporting information can be downloaded at: https://www.mdpi.com/article/10.3390/microorganisms12071424/s1, Video S1: Case 3— Circling; Video S2: Case 5— Circling.
